# Refilins are short-lived Actin-bundling proteins that regulate lamellipodium protrusion dynamics

**DOI:** 10.1242/bio.019588

**Published:** 2016-10-15

**Authors:** Olivia Gay, Benoît Gilquin, Nicole Assard, Pascal Stuelsatz, Christian Delphin, Joël Lachuer, Xavier Gidrol, Jacques Baudier

**Affiliations:** 1 INSERM U873 and INSERM Unité 1038, Grenoble F-38000, France; 2 CEA, BIG, BGE, Grenoble F-38000, France; 3 Université Grenoble Alpes, Grenoble F-38000, France; 4 Genomic and Microgenomic Platform, ProfileXpert, Bron F-69676, France; 5 Lyon Neuroscience Research Center INSERM U1028/CNRS UMR 5292, Lyon F-69372, France

**Keywords:** FilaminA, Polydendrocyte, RefilinA, RefilinB, FAM101A, FAM101B

## Abstract

Refilins (RefilinA and RefilinB) are members of a novel family of Filamin binding proteins that function as molecular switches to conformationally alter the Actin filament network into bundles. We show here that Refilins are extremely labile proteins. An N-terminal PEST/DSG(X)_2-4_S motif mediates ubiquitin-independent rapid degradation. A second degradation signal is localized within the C-terminus. Only RefilinB is protected from rapid degradation by an auto-inhibitory domain that masks the PEST/DSG(X)_2-4_S motif. Dual regulation of RefilinA and RefilinB stability was confirmed in rat brain NG2 precursor cells (polydendrocyte). Using loss- and gain-of-function approaches we show that in these cells, and in U373MG cells, Refilins contribute to the dynamics of lamellipodium protrusion by catalysing Actin bundle formation within the lamella Actin network. These studies extend the Actin bundling function of the Refilin-Filamin complex to dynamic regulation of cell membrane remodelling.

## Abstract (precis)

**Summary:** The newly identified Refilin protein family are unique short-lived proteins that complex with Filamin to regulate plasma membrane dynamics though the promotion of Actin filament bundling.

## INTRODUCTION

Actin assembly and dynamics are vitally important for cell-cell interactions and cell motility from metastatic tumour cells to the growing tips of neuronal growth cone. In migrating cells and in growth cones, various higher-order networks of Actin filaments are used to power motility, making the identification and characterization of specific Actin networks and regulatory proteins a priority for understanding cell motility in general (He et al., 2015; Hotulainen and Hoogenraad, 2010; Pak et al., 2008).

The Refilins (RefilinA and RefilinB) are a newly identified family of Actin-bundling proteins. In cells Refilins interact with the Filamin family of Actin-binding proteins (FLNA, FLNB, FLNC) and convert Filamins from Actin branching into Actin-bundling proteins (Gay et al., 2011a,b,c). In epithelial cells, RefilinB, whose expression depends on TGF-β stimulation, functions as a regulator of apical perinuclear Actin reorganization in early steps of epithelial mesenchymal transition (EMT) (Gay et al., 2011b,c). In fibroblast, RefilinB expression depends on confluence and promotes the recruitment of FLNA on perinuclear Actin filament bundles forming an Actin cap. Filamins integrate cellular architectural and signalling parameters and are essential for normal fetal development (Feng and Walsh, 2004; Popowicz et al., 2006; Stossel et al., 2001; Zhou et al., 2010). Deleterious mutations in Filamin genes cause a wide range of muscular dystrophies and developmental malformations in the heart, skeleton and brain (Krakow et al., 2004; Lu et al., 2007; Robertson et al., 2003). Mice with loss of a single *Refilin* (*Cfm*, *FAM101*) gene displayed no overt phenotype (Hirano et al., 2005; Mizuhashi et al., 2014), whereas *Refilin* double-knockout (KO) mice showed skeletal malformations resembling those of FilaminB (FLNB)-deficient mice (Mizuhashi et al., 2014). This points to functional redundancies between RefilinB (also called Cfm1 or FAM101B) and RefilinA (also called Cfm2 or FAM101A) and suggests essential functions of the Refilin/Filamin complex during embryonic development. It is now of particular interest to understand the cellular regulations and functions of the Refilin/Filamin complex.

Here, we show that Refilins are extremely labile proteins and that different mechanisms control RefilinA and RefilinB levels in rat brain NG2 cells. NG2 cells, also referred to as oligodendrocyte precursor cells (OPCs) or polydendrocytes, represent a major resident glial cell population that is distinct from mature astrocytes, oligodendrocytes, microglia, and neural stem cells and exist throughout the grey and white matter of the developing and mature central nervous system (CNS) (Nishiyama et al., 2015, 2009). In NG2 cells, RefilinA level depends on transcriptional regulation whereas RefilinB level relies on increased protein stability. In these cells, Refilins contribute to the dynamics of lamellipodium protrusion. These studies extend the function of the Refilin/Filamin complex to regulation of Actin assembly and dynamics for cell membrane remodelling.

## RESULTS

### RefilinA and RefilinB are short-lived proteins

**Fig. 1.**
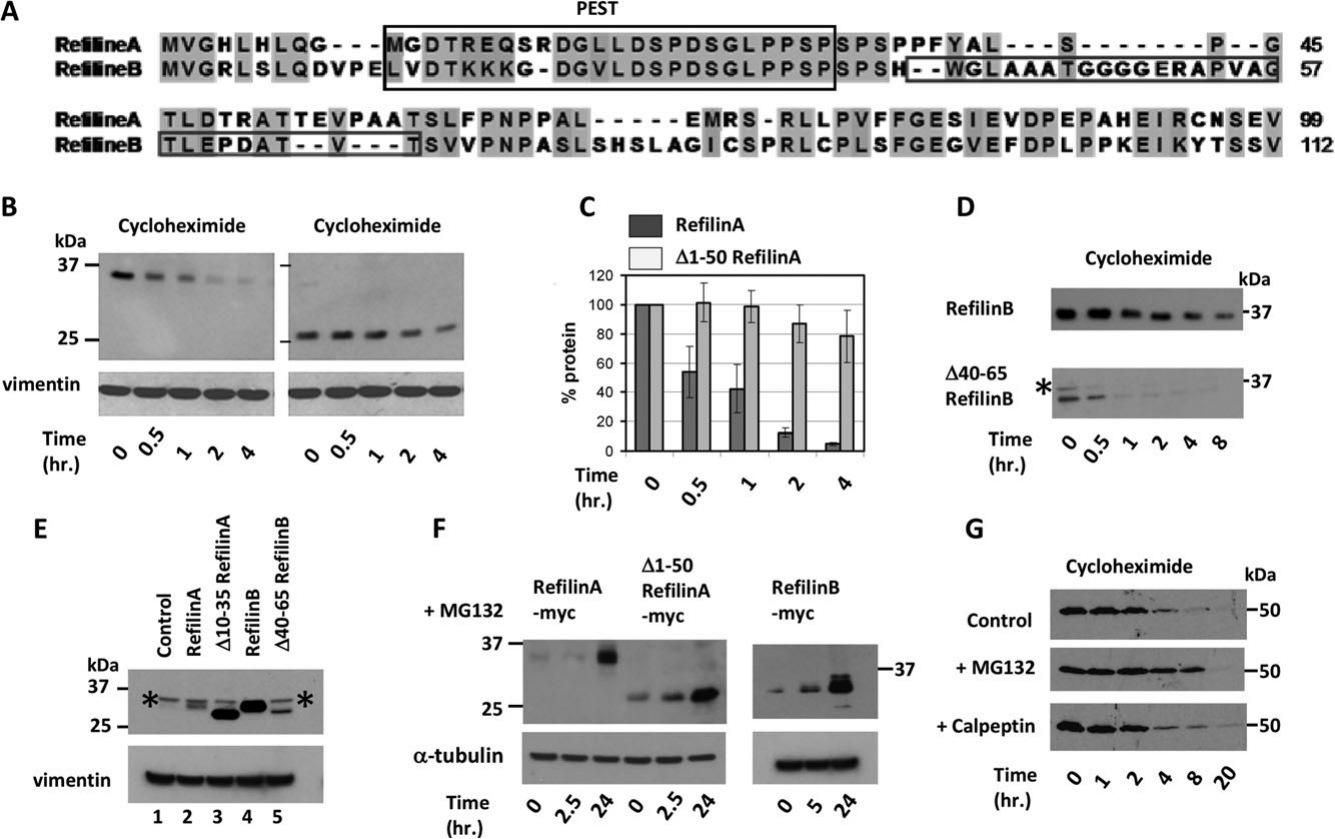
**Refilins are short-lived proteins.** (A) Sequence alignment of the N-terminus of rat RefilinA (residues 1-99) and RefilinB (residues 1-112) proteins show conserved N-terminal sequence harbouring a PEST/DSG(X)_2-4_S motif (PEST). The specific adjacent sequence only found in RefilinB is squared. (B,C) Cycloheximide chase analysis of full­-length and truncated 50-204 Myc-tagged RefilinA proteins expressed in sub-confluent U373MG cells. Transfected cells were incubated with cycloheximide (100 µg/ml). Cell extracts were resolved on 12% SDS-PAGE and analysed by western blot using chicken anti-RefilinA or mouse anti-Vimentin as loading control. (C) Quantitative analysis of the western blot shown in panel B of two independent cycloxeximide chase experiments. The mean±standard error (s.e.m.) of two different experiments are shown, statistically different from control condition (Student's *t*-test, *P*<0.009). (D) Sub-confluent U373MG cells transfected with Myc-tagged RefilinB (upper panel) and truncated Δ40-65 RefilinB (lower panel). After 20 h cells were incubated with cycloheximide (100 µg/ml) for the indicated times. Cell extracts were analysed by western blot using mouse anti-Myc monoclonal antibody. Asterisks point to a non-specific band specifically recognized by the mouse anti-Myc monoclonal antibody (see also E,F). (E) Comparison of the steady-state level of Myc-tagged RefilinA (lane 2), Myc-tagged truncated Δ10-35-RefilinA (lane 3), Myc-tagged RefilinB (lane 4), and Myc-tagged truncated Δ40-65-RefilinB (lane 5) expressed in U373MG cells. Sub-confluent U373MG cells in 100 mm plates were transfected with 2 µg of recombinant pcDNA-3 plasmids and incubated 20 h prior analyses. Lane 1 is a non-transfected control cell lysate. Cell extracts were analysed by western blot using anti-Myc. Asterisks point to a non-specific band specifically recognized by the mouse anti-Myc monoclonal antibody. (F) Sub-confluent U373MG cells were transfected with Myc-tagged RefilinA, truncated 50-204 RefilinA (Δ1-50) and RefilinB constructs as indicated. After 20 h, transfected cells were incubated with protease inhibitor MG132 (5 µM) for the indicated times. Cell extracts were analysed by western blot using rat anti-Myc and anti-α-tubulin. The non-specific band recognized by the mouse anti-Myc monoclonal antibody (* in D,E) is not recognized by the rat anti-Myc antibody. (G) Sub-confluent U373MG cells transfected with RefilinA-GFP were incubated with cycloheximide (100 µg/ml) in the absence or in the presence of MG132 or Calpeptin for the indicated times. Total cell extracts were analysed by western blot using mouse anti-GFP antibody.

Sequence analysis reveals that the N-termini of Refilins are characterized by two overlapping degradation signals: a conserved PEST degradation signal (Pestfind score: 7.8 and 10.2 for RefilinA and B, respectively) and a DSG(X)_2-4_S motif that promotes the rapid degradation of short-lived proteins (Fig. 1A) (see also Busino et al., 2003; Gay et al., 2011a; Suzuki et al., 2010; Zhou et al., 2004). To study Refilin degradation we transfected U373 MG cells that do not express endogenous Refilin with various RefilinA-Myc, RefilinA-GFP or RefilinB-Myc expression plasmids. Combining cycloheximide chase and western blot analyses, the half-life of recombinant RefilinA-Myc fusion proteins was between 30 min and 1 h (Fig. 1B,C). The half-life of RefilinB-Myc was significantly longer, ranging from 2 h to 8 h depending on the cell density (Fig. 1D). The effect of cell density on Refilin stability has been previously reported (Gay et al., 2011b). Deletion of the 50 N-terminal amino acids. (Fig. 1B,C) or selective removal of the PEST/DSG(X)_2-4_S motif (residues 10-35) from RefilinA increased the half-life of the truncated proteins, although mutant proteins were still subjected to subsequent degradation (Fig. 1B,C). As a consequence of these deletions, the steady-state level of the Δ10-35-RefilinA-Myc protein became similar to that of RefilinB-Myc (Fig. 1E, lanes 3 and 4).

To explain the higher stability of RefilinB-Myc, we have identified a sequence present only in RefilinB (residues 40-65), contiguous to the PEST sequence, that functions as an auto-inhibitory domain for the PEST/DSG(X)_2-4_S degradation signal (Fig. 1A). Deletion of this sequence resulted in a dramatic decrease of the Δ40-65-RefilinB-Myc mutant protein half-life (Fig. 1D) and a steady state level similar to that of RefilinA (Fig. 1E, lanes 2 and 5).

As seen with other short-lived proteins bearing DSG(X)_2-4_S motifs (Busino et al., 2003; Zhou et al., 2004), a dramatic increase in both RefilinA and RefilinB levels was observed upon incubation of the transfected cells with the cell permeable proteasome inhibitor MG132 (Fig. 1F). However, stabilization of Refilins occurs only after long-term incubation with MG132. Remarkably, the Δ50-RefilinA lacking the PEST/DSG(X)_2-4_S motif was also protected from degradation by MG132 to the same extent as the full length RefilinA (Fig. 1F). If MG132 is present during the cycloheximide chase, the degradation of RefilinA-GFP is delayed but not inhibited (Fig. 1G). The Calpain-specific inhibitor Calpeptin, which protects some proteins with PEST sequence against degradation, did not stabilize RefilinA-GFP (Fig. 1G). Taken together, these data suggest the PEST/DSG(X)_2-4_S motif functions in rapid degradation in a proteasome-independent manner and that another domain within the C-terminus of Refilins is also a target for slow degradation (see Discussion section).

### Interactions with Filamin protects Refilins from proteasomal degradation

**Fig. 2.**
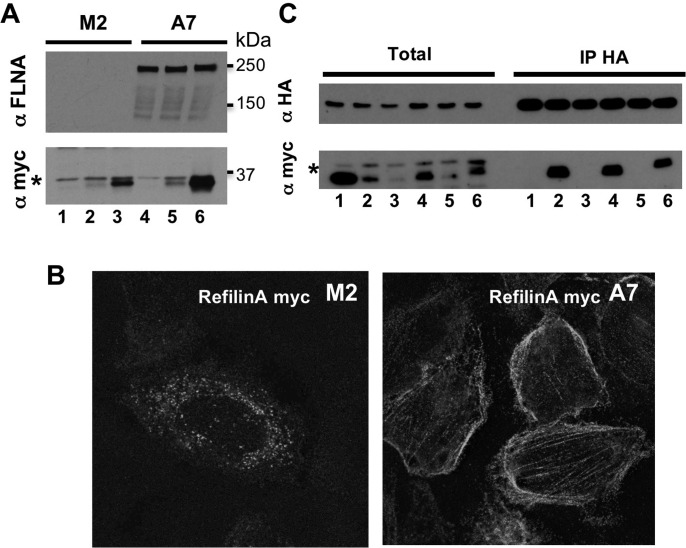
**The interaction with Filamin protects Refilins from degradation.** (A) Sub-confluent FLNA-deficient M2 cells (lanes 1-3) and A7 cells (lanes 4-6) were not transfected (lanes 1,4) or transfected with Myc-tagged RefilinA (lanes 2,5) or RefilinB (lanes 3,6). Total cell extracts were analysed by western blot with anti-Myc and anti-FLNA antibodies. (B) M2 cells (left panel) and A7 cells (right panel) transfected with Myc-tagged RefilinA were fixed and stained with anti-Myc antibodies. (C) Sub-confluent FLNA-deficient M2 cells co-transfected with 19-22 FLNA-HA and either Myc-tagged control protein (Miret) (lane 1), RefilinA-ΔBD_1_ to -ΔBD_4_ mutants (lanes 2-5) or wild-type RefilinA (lane 6) were immunoprecipitated with rat anti-HA antibody. Total cell extracts (Total) and immunoprecipitates (IP-HA) were analysed by western blot using mouse anti-HA and mouse anti-Myc. In A and C, asterisks indicate to a non-specific band.

In the cells, Refilins interact with Filamin. To examine the role of this interaction in the control of Refilin stability, we first compared the steady state levels of transfected RefilinA and RefilinB in A7 and M2 cell lines that do or do not express Filamin. In M2 cells, RefilinA is almost undetectable and the steady state level of RefilinB is very low (Fig. 2A, lanes 1-3). Indirect immunofluorescence analysis shows that in transfected M2 cells, RefilinA immunoreactivity is hardly visible and appears as punctate dots in the cytoplasm (Fig. 2B, left panel). In A7 cells Refilins levels increase (Fig. 2A, lanes 4-6) and the proteins co-localize with Filamin on Actin stress fibres (Fig. 2B, right panel) (see also Gay et al., 2011b). To conclusively demonstrate that the interaction with FLNA contributes to RefilinA stabilisation, RefilinA or individual binding domain (BD) deletion mutants RefilinA-ΔBD_1_-Myc through RefilinA-ΔBD_4_-Myc were co-transfected with the FLNA binding domain 19-22-HA construct in M2 cells (Gay et al., 2011b). The FLNA 19-22-HA was then immunoprecipitated with anti-HA antibodies and total cellular extract and immunoprecipitates were analysed by western blot for RefilinA presence (Fig. 2C). Only RefilinA and RefilinA-ΔBD_1_ and -ΔBD_3_ mutants that had the ability to interact with 19-22-FLNA-HA were stabilized and showed high steady state level in total cell extracts.

### Dual regulation of RefilinA and RefilinB proteins in rat brain NG2 precursor cells

**Fig. 3.**
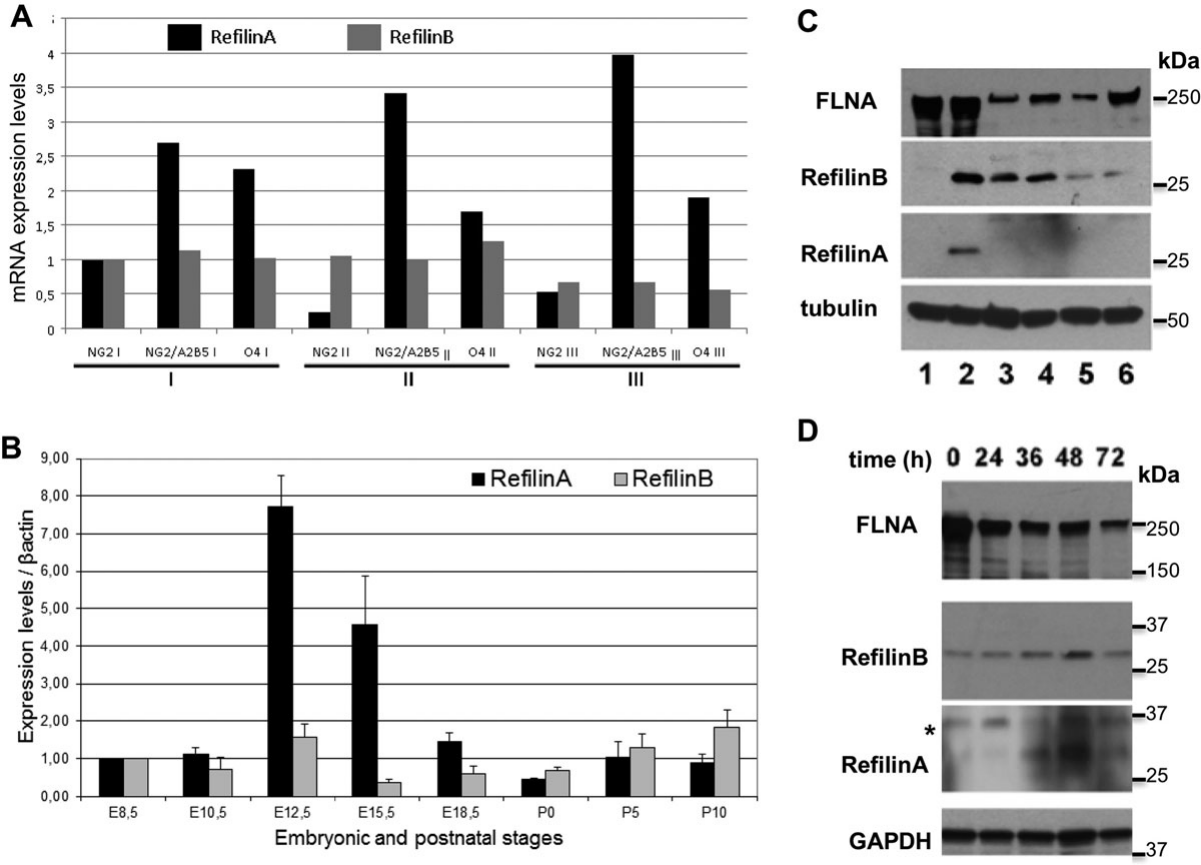
**Differential regulation of RefilinA and RefilinB mRNAs and proteins.** (A) Comparison of RefilinA and RefilinB mRNA levels in three cell sorting experiments (see Fig. S1C). (B) Comparison of RefilinA and RefilinB mRNA levels during mouse brain development. 1 µg of cDNA from embryonic and postnatal mouse brain [E8.5; E10.5; E12.5; E15.5; E18.5; postnatal day (P)0; P5; P10] were used to perform quantitative PCR. *β-actin* gene was selected as stable reference gene in embryo for internal standardization (Willems et al., 2006). Error bars represent the standard deviation (s.d.) of three independent experiments. We assigned the enrichment value of 1 for stage E8.5. (C) Western blot analyses of total cellular extracts of U373MG cells (lane 1), U373MG cells transfected with RefilinB or RefilinA constructs (lane 2), long term rat OPC cultures (lanes 3,5) or rat neural progenitors (lanes 4,6). OPC and neural progenitors were grown in Neurobasal medium supplemented with bFGF and PDGF (lanes 3,4) or bFGF and EGF (lanes 5,6). Anti-FLNA, anti-RefilinB, anti-RefilinA and anti-β-Tubulin antibodies were used as indicated. (D) Rat OPC cultures were shifted to differentiation culture medium for different period of time as indicated and lysed in SDS-sampling buffer. Total cell extracts (20 µg) were analysed by western blot using anti-FLNA, anti-RefilinB, and anti-GAPDH antibodies. A fivefold greater concentration of loaded sample was required to detect RefilinA (100 µg) with anti-RefilinA antibody.

Oligodendrocyte precursor cells purified from newborn rat brain can be expanded *in vitro* in the presence of bFGF and PDGF for several months (Tang et al., 2000). The cultures contain a mixture of amplifying cells in distinctive stages of differentiation characterized by selective expression of cell surface antigens (Fig. S1A,B). Cells characterized by the exclusive expression of the proteoglycan marker NG2 are considered multipotent precursor cells. These cells do express the neural stem cell marker Nestin (not shown) and can evolve into oligodendrocyte precursor cells (OPC) that co-express NG2 and the early OPC marker A2B5 (NG2+/A2B5+) (Fig. S1A,B). In these cultures, few cells are NG2 negative and express the late OPC differentiation marker O4 (O4+) (Fig. S1A,B). Comparative transcriptomic analysis between the three cell populations identified RefilinA mRNA (*FAM101A*) which is specifically upregulated in NG2+/A2B5+ cells (Fig. S1C,D). The complete list of regulated genes during transition of NG2+ cells into NG2+/A2B5+ cells is reported in Table S1. Increased mRNA levels of RefilinA in NG2 cells was confirmed by RT-PCR (Fig. 3A). The increase in RefilinA mRNA appears specific and is not observed with RefilinB (FAM101B, Cfm1) mRNA (Fig. 3A). Differential mRNA levels of RefilinA versus RefilinB was also confirmed on whole tissues analyses (Fig. 3B) where only RefilinA mRNA showed high variation during early mouse brain development. This timing of RefilinA (Cfm2) expression in the brain is consistent with a previous *in situ* hybridisation study (Hirano et al., 2005).

We next compared RefilinA and RefilinB protein expression in amplifying rat NG2 precursor cells by western blot analyses (Fig. 3C). To verify the identities of the Refilin bands, we used U373MG cell extracts from non-transfected cells, (lane 1) or cells transfected with a mixture of recombinant plasmids expressing wild-type rat RefilinA and RefilinB species (lane 2). Both NG2+ amplifying rat oligodendrocyte precursor cells (lanes 3 and 5) and NG2+ rat neural progenitor cells grown as neurospheres (Balenci et al., 2006) (lanes 4 and 6) were analysed. Only RefilinB is detectable in NG2 precursor cell extracts (lanes 3-6). RefilinA is below the detection limit (lanes 3-6). The expression of RefilinB is dependent upon PDGF stimulation; cellular RefilinB content decreases when PDGF is replaced with EGF (lanes 5 and 6). PDGF favours oligodendrocyte lineage differentiation (Tang et al., 2000), whereas EGF maintains multipotent differentiation potential (Doetsch et al., 2002).

**Fig. 4.**
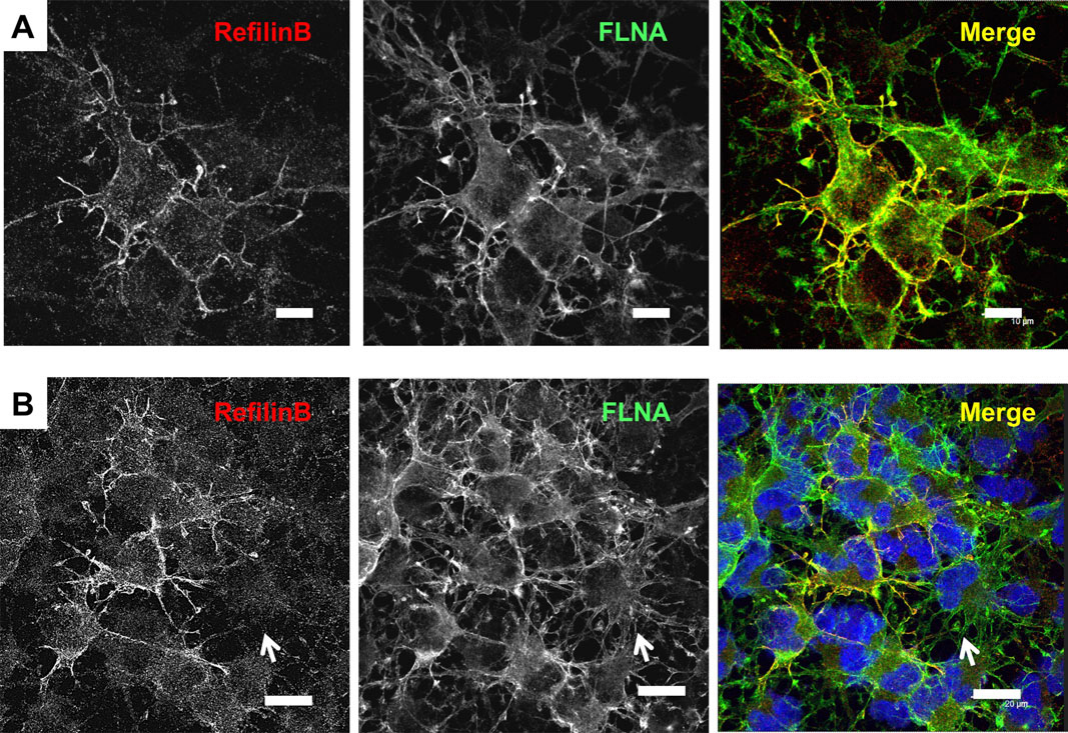
**RefilinB co-localizes with FLNA at the plasma membrane and membrane protrusions in NG2 oligodendrocyte precursor cells.** (A,B) High-density OPC cultures were fixed in methanol and double immunostained with guinea pig anti-RefilinB (red) and mouse anti-FLNA (green) antibodies. In B, nuclei were stained with Hoescht. Scale bar: 10 µm (A) and 20 µm (B). In B, arrows point to cell with no RefilinB immunostaining.

We next analysed the steady state levels of RefilinA and RefilinB during the maturation of oligodendrocyte precursor cells (Fig. 3B). When shifted to differentiation culture medium, oligodendrocyte precursor cells underwent progressive immunological and morphological transformation (Fig. S2A-C). After 72 h, cells completely lose NG2 antigen and express the late OPC differentiation marker O4 (Fig. S2A-C). The transition from NG2+ precursors into O4+ OPC correlates with morphological changes. O4+ cells are characterized by irregularly shaped cell bodies from which multiple fine branching processes radiate (Fig. S2A,B). Consistent with the genomic analysis, a transient increase in RefilinA protein can be observed with maximal expression preceding that of the late differentiation marker O4 (Fig. 3B). However, the RefilinA band is only visible after overloading protein extract samples and overexposure of the Western blot membrane allowed when compared to RefilinB. Both RefilinA and RefilinB protein content decreased in O4+ cell extracts. The decrease in RefilinB levels in morphologically differentiated cells was confirmed by indirect immunofluorescence analysis (see Fig. 4B).

Taken together, these results show that Refilin expression is specific to NG2+ cells committed to the oligodendrocyte lineage. They also point to dual regulation of Refilins in NG2 cells. RefilinA levels are regulated at the mRNA level, whereas RefilinB level rely on higher intrinsic protein stability.

### Refilins co-localize with Filamin on lamellipodial protrusions in NG2 precursor cells

Due to the very low expression level of RefilinA in NG2 cells, it was not possible to examine the cellular localization of the endogenously expressed RefilinA protein in these cells. For the examination of RefilinB, we used methanol fixation to minimize nonspecific background staining. Moreover, cells were grown to confluence to increase RefilinB immunostaining. Under these conditions, RefilinB immunoreactivity localizes with FLNA along the membranes of dendritic processes and filopodia protrusions (Fig. 4A). Variability in RefilinB immunostaining intensity is observed between cells. RefilinB immunoreactivity is high in cells that have not yet developed a multi-branched morphology and is downregulated in cells characterized by large arborized network of cellular processes typical of NG2−/O4+ OPC (Fig. 4B, arrow).

**Fig. 5.**
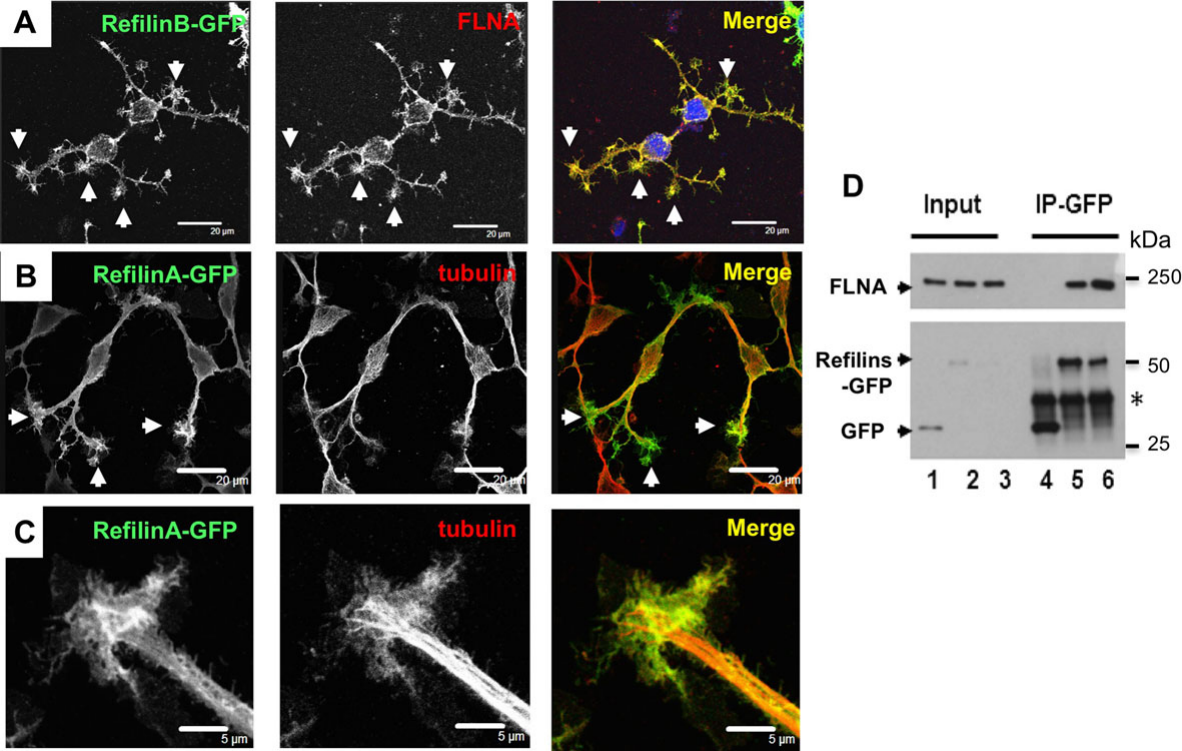
**RefilinA-GFP and RefilinB-GFP accumulate on lamellipodia protrusion in NG2 cells.** (A-C) Low-density OPC cultures were infected with recombinant adenovirus expressing RefilinB-GFP (A) or RefilinA-GFP (B,C). After 24 h, cells were fixed in methanol (A) or PFA (B,C), and double immunostained with rabbit anti-GFP antibodies (A-C, green) and mouse anti-FLNA antibodies (A, red) or mouse anti-β-tubulin (B,C; red). We use anti-GFP antibodies to enhance Refilins-GFP staining intensity. Cells were observed by confocal microscopy. Arrows point to lamellipodia and growth cone-like structures. In C, high magnification of a lamellipodial growth cone is shown. Scale bar: 20 µm (A,B) and 5 µm (C). (D) FLNA is a major target for Refilins-GFP in oligodendrocyte precursor cells. OPC cultures infected with recombinant adenovirus expressing GFP-control (lanes 1), GFP-RefilinB (lane 2) or GFP-RefilinA (lanes 3) were used for immunoprecipitation with rabbit anti-GFP antibody. Total cell extracts (Input) and immunoprecipitates (IP-GFP) were resolved on 6% and 12% SDS-PAGE gels and analysed by western blot using anti-FLNA or mouse anti-GFP antibodies, respectively. Asterisk indicates position of the light chain IgG.

In cells infected with recombinant adenovirus expressing RefilinB-GFP (Fig. 5A) or RefilinA-GFP (Fig. 5B,C), the GFP fusion proteins accumulated along the plasma membrane and on lamellipodial protrusions that form along or at the tips of growing processes (white arrows). The Refilin proteins co-localize with FLNA (Fig. 5A). At the tips of growing processes, the structures labelled with RefilinA-GFP have characteristics of lamellipodial growth cones (Fig. 5C). Within these structures RefilinA-GFP complexes accumulate in the transitional zones where parallel microtubule bundles become defasciculated and extend individually into the proximal domain. There is no evidence for filopodia labelling by RefilinA-GFP. Co-IP studies confirmed that FLNA is a major target for RefilinB-GFP and RefilinA-GFP in infected OPC (Fig. 5D).

### RefilinB regulates lamellipodia dynamics in NG2 precursor cells

The accumulation of the Refilin-GFPs and FLNA in lamellipodial growth cones prompted us to investigate whether the Refilin/FLNA complex functions in lamellipodia dynamics. Lamellipodia are very delicate structures which are damaged upon fixation; consequently they are best seen in studies that use live imaging (Wood and Martin, 2002). We used loss- and gain-of-function approaches targeting RefilinB, because it is the only Refilin species constitutively expressed in amplifying NG2 precursor cells (Fig. 3C).

**Fig. 6.**
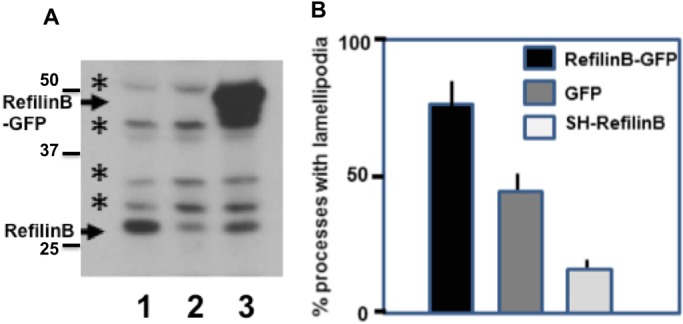
**RefilinB regulates the dynamic of lamellipodia in NG2 cells.** (A) OPC were infected with recombinant adenovirus expressing GFP-control (lane 1), ShRefilinB (lane 2) or RefilinB-GFP (lane 3) and grown for 24 h in differentiation culture medium. Total cell extracts were analysed by western blot using anti-RefilinB antibodies. Arrows point to specific RefilinB immunoreactivities, asterisks indicate non-specific bands. (B) Video recordings of OPC infected with recombinant adenovirus expressing GFP-control (GFP), ShRefilinB or RefilinB-GFP were used to quantify the percentage of processes that form lamellipodia during the time of recording (10 min). The results are the average of two infection experiments and the analyses of 100 processes per experiment. Error bars represent the variability of data between two independent experiments

Amplifying NG2 precursor cells grown in the presence of bFGF and PDGF were infected with recombinant adenoviruses expressing GFP, RefilinB-GFP fusion protein, or shRNAs directed against RefilinB, and then grown in the presence of PDGF alone. The adenovirus infection had nearly 100% efficiency and was non-toxic for cells. The inhibition of RefilinB expression by shRNA and upregulation of RefilinB-GFP in infected OPC was confirmed by western blot analysis (Fig. 6A). Virally transduced cells were observed by time-lapse phase contrast imaging (Movies 1-4). We controlled the expression of GFP or Refilin-GFP fluorescence prior to recording. In cells expressing GFP, dynamic lamellipodium protrusions transiently form and collapse at the tips of dendritic processes (Movies 1 and 2, left panels). In cells overexpressing RefilinB-GFP, lamellipodia are much more visible mostly due to an increase of the number of lamellipodium protrusions, an increase of the lamellipodium veil surface and an increase in lifetime of the newly formed lamellipodia (Movie 2, right panel and Movie 3, left panel). Suppression of RefilinB by shRNA decreases the frequency at which lamellipodia form at the tips and along the growing processes (Movie 1, 3 and 4, right panels). The contribution of RefilinB to lamellipodial dynamics was confirmed by quantitative analysis of the percentage of processes that produced lamellipodium veils in cells expressing GFP-RefilinB, control GFP or shRNA against RefilinB during the recording time (Fig. 6B).

### RefilinA regulates the construction of Actin bundles in the lamella of spreading U373MG cells

**Fig. 7.**
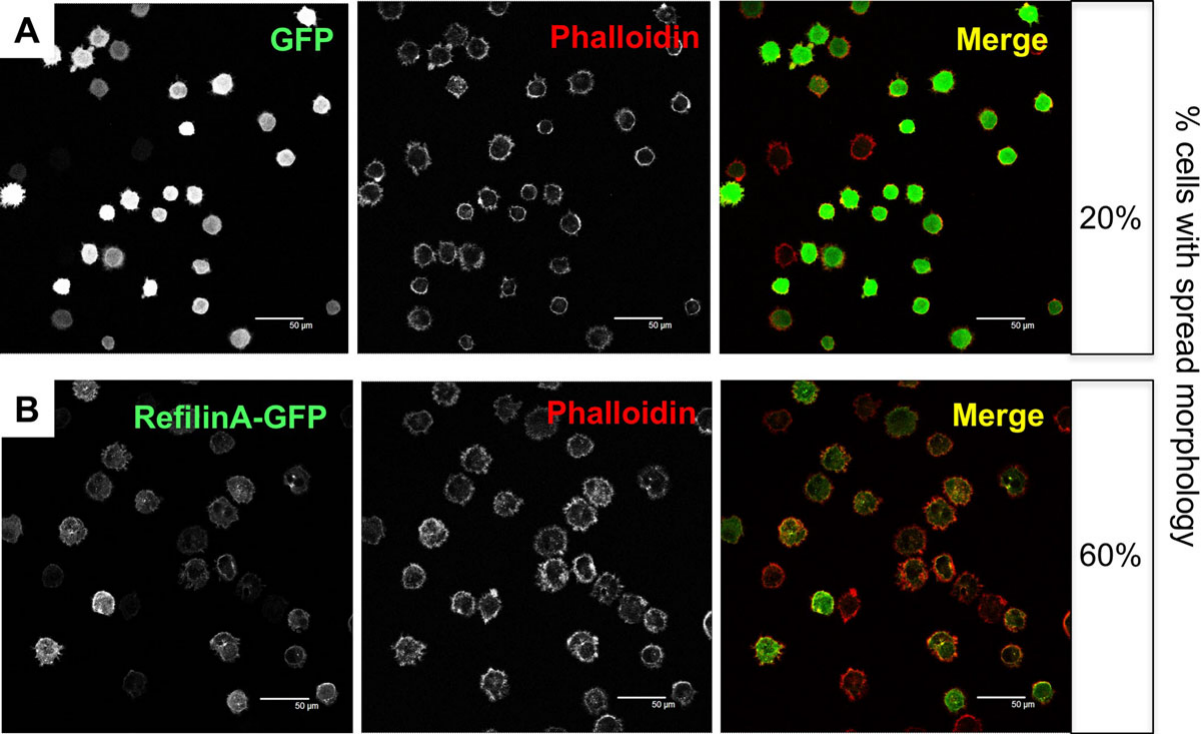
**RefilinA enhances spreading of U373MG cells.** U373MG cells were infected with recombinant adenovirus expressing GFP-control (A) or GFP-RefilinA (B). After 24 h, cell spreading was quantified by allowing the cells to spread on fibronectin-coated coverslips for 20 min. Cells attached to coverslips were fixed with 4% formaldehyde in phosphate-buffered saline (PBS) for 10 min and permeabilized with 0.2% Triton X-100 in PBS for 3 min. Cells were blocked with 3% BSA in PBS containing 0.1% Tween 20, and incubated with Alexa Fluor 486 Phalloidin for 1 h. Cells were mounted and visualized under a confocal microscope at low magnification (A,B). Scale bar: 50 µm. On the right side the percentage of cells adopting spread morphology is reported. Non-spread cells were defined as round cells, whereas spread cells were defined as those that lacked a rounded shape and had extended membrane protrusions. In each experiment, more than 150 cells were counted. Two independent experiments were performed.

**Fig. 8.**
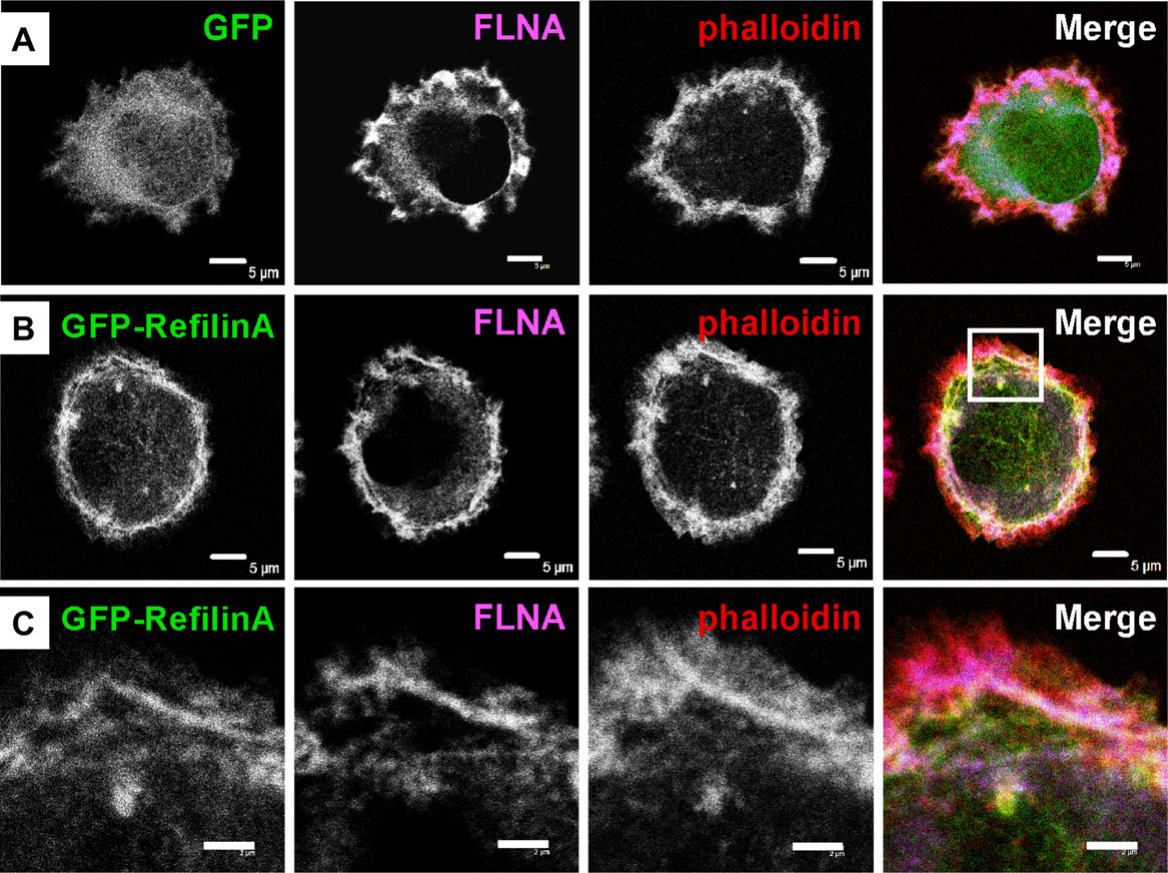
**RefilinA contributes to the construction of Actin bundles in the lamella of spreading U373MG cells.** Confocal microscope observations of spreading U373MG cell infected with recombinant adenovirus expressing GFP-control (A) or GFP-RefilinA (B,C) stained with Alexa Fluor 486 Phalloidin and immunostained with FLNA antibody (blue). Panel C shows enlargement of the squared area shown in B. Scale bar: 5 µm (A,B) and 2 µm (C).

A direct contribution of Refilin to lamellipodial dynamics was confirmed by analysing spreading of U373MG cells infected with recombinant adenovirus expressing GFP-control or GFP-RefilinA on Fibronectin-coated coverslips (Figs 7, 8). Two regions define membrane protrusion of cell spreading, the lamellipodium consisting mostly of reticulated Actin network, and the lamella, found immediately behind the lamellipodium and composed of Actin arc bundles parallel to the edge (Burnette et al., 2011).

When infected with recombinant adenovirus-expressing RefilinA-GFP, U373MG cells showed more rapid spreading on Fibronectin-coated dishes than those infected with control adenovirus (Fig. 7A-B). In spreading U373MG cells, RefilinA-GFP accumulated at the cell periphery (Fig 7B, Fig 8B) and re-localizes FLNA from diffuse patches (Fig. 8A) onto bundles of Actin arcs localized behind the lamellipodium Actin meshwork (Fig. 8B,C). As previously reported for perinuclear Actin bundles mediated by Refilins in confluent U373A cells (Gay et al., 2011b), the Actin bundles decorated by RefilinA-GFP are immunostained with anti-Myosin II antibody, indicating that they are contractile Actin bundles (not shown).

These results strongly suggest that Refilins function in spreading U373MG cells as switches to convert Filamins from Actin branching into Actin bundling proteins to structure Actin arcs in the lamella that form from the Actin meshwork at the cell edge to enhance lamellipodial protrusion (Nemethova et al., 2008; Burnette et al., 2011).

## DISCUSSION

### Refilins are short-lived proteins; biological implications

Refilins (RefilinA and RefilinB) comprise a novel family of Actin regulatory proteins that function as molecular switches to interconvert the Actin meshwork into bundles (Gay et al., 2011b). One remarkable property of this new family of regulatory protein is their short half-life, which is unique among Actin regulatory proteins. Although interactions with FLNA protect Refilins from degradation to some extent, RefilinA still has an extremely short half-life which makes the protein weakly detectable using conventional immunological tools. The high turnover of Refilins is mediated by conserved PEST degradation signals which overlap with a DSG(X)_2-4_S motif. In addition, a second degradation signal localizes within the C-terminus, suggesting a two-step degradation process for Refilins. Both wild-type RefilinA and the Δ50-RefilinA lacking the PEST/DSG(X)_2-4_S motif are stabilized when cells are treated with the cell-permeable proteasome inhibitor MG132 (Fig. 1F). Stabilization of Refilins is observed only after long-term incubation with MG132, whereas stabilization of protein degraded strictly in a proteasome-dependent manner occurs within minutes after the addition of MG132 (Mathes et al., 2010; Zhou et al., 2004). Several attempts to demonstrate a physical association of Refilins with βTrcp that recognizes the DSG(X)_2-4_S motif for proteasome-dependent protein degradation (Zhou et al., 2004) were unsuccessful (data not shown). Ubiquitin-independent degradation of RefilinA was confirmed by mutagenesis studies. Mutations of the only two lysine residues present in rat RefilinA (K128 and K179) have no effect on the protein half-life (data not shown). Together, these data point to a complex ubiquitin-independent degradation pathway for Refilins. Refilin degradation shows many similarities with that of IκBα (Mathes et al., 2008, 2010). Like Refilins, IκBα has both a PEST and a DSG(X)_2-4_S degradation motif and PEST-mediated degradation of IκBα is ubiquitin-independent. Moreover, the PEST domain of IκBα is protected from proteasomal recognition by interaction with neighbouring sequences and interactions between IκBα and its target, NF-kB. As proposed for IκBα (Mathes et al., 2010), the presence of N-terminal and C-terminal degradation domains on Refilins illustrate the need for complete fragmentation of the proteins for functional inactivation. Switching FLNA from an Actin-branching protein into one that bundles is a two­-step processes requiring Refilin dimerization through its N-terminal region and interaction of the C-terminal peptide regions of the Refilin dimers with FLNA (Gay et al., 2011b). When fused to GFP (N-terminal or C-terminal), the minimal Refilin region required to interact with FLNA (residues 128-175 on RefilinA) was equally as potent as full length RefilinA in co-immunoprecipitating FLNA (Fig. S3A) and contains all the information necessary to promote the re-localization of FLNA on Actin stress fibres (Fig. S3B). Thus both N-terminus and C-terminus fragmentation are required for full inactivation of Refilins when bound to FLNA.

Several functional characteristics may explain why Refilin is rapidly degraded when bound to Filamin. In cells, Filamin binds all Actin isoforms (e.g. F-Actin, G-Actin) and its structural organization allows it to form a flexible bridge between two Actin filaments at various angles, thereby imparting the Actin network with loose or gel-like qualities. Filamin also simultaneously interacts with and influences the activity of a number of other diverse proteins (e.g. transmembrane receptors, cell adhesion molecules, signalling molecules) (Feng and Walsh, 2004; Popowicz et al., 2006; Stossel et al. 2001; Zhou et al. 2010). None of the partner binding interactions alter Filamin-Actin binding. Refilins are the only Filamin-binding partners that can profoundly modify the mechanism of Filamin crosslinking by switching Filamins from Actin-branching into Actin-bundling proteins. The Actin-bundling activity mediated by the Refilins is likely to produce changes not only in Actin organization, but also in the interaction of Filamin with other partners. Hence, interactions between Refilins and Filamins must be fully reversible to preserve the dynamics of the Actin cytoskeleton and the Filamin regulatory functions. Refilin degradation is likely a key mode of negative regulation of the bundling activity mediated by the Refilin/Filamin complex. To support this model, we have found that in a derivative clone of the U373MG cell line, the U373A cells selected for its tumorigenicity, the degradation of RefilinA is markedly decreased compared to that in the parental U373MG cell line (Fig. S4A). As a consequence, the steady state level of transfected RefilinA is much higher and promotes formation of Actin superstructures (Gay et al., 2011b) (see also Fig. S4b). Confocal microscopy and 3D reconstitution reveals that the Actin superstructure mediated by the RefilinA in U373A cells are composed of thick density enriched in F-Actin that connect the apical and basal membranes from which star-shaped Actin stress fibres project at the ventral surface (Fig. S4C). Similar star-shaped Actin bundles have previously been observed in cells that were manipulated to increase Actin bundles by the depletion of Actin capping protein (Mejillano et al., 2004).

### Refilin regulates membrane dynamics

To balance rapid, specific protein degradation, cells can control Refilins levels through at least two different mechanisms. We have shown that in NG2 cells, RefilinA expression is regulated at the steady state mRNA level, whereas RefilinB levels rely on higher intrinsic protein stability. NG2 cells are extremely sensitive to cycloheximide and MG132 treatment and could not be cultured in the presence of the drugs. Consequently, we were not able to determine if degradation is solely responsible for the extremely low RefilinA level in these cells. In NG2 cells, RefilinA and RefilinB expression is specific to cells committed to the oligodendrocyte lineage (NG2+/A2B5+) but is downregulated in post-mitotic cells characterized by expression of the O4 antigen (NG2−/O4+). This suggests that Refilins are not associated with oligodendrocyte differentiation. Zebrafish represent a suitable animal model to study oligodendrocyte differentiation *in vivo* (Kirby et al., 2006). Considering that a single *Refilin* (*FAM101*) gene is present in zebrafish, the effect of silencing Refilin on zebrafish OPC differentiation was tested. No effect of silencing Refilin on zebrafish oligodendrocyte development was observed. Oligodendrocytes formed in normal numbers, migrated to, and wrapped axons normally (Dr. Bruce Appel, Anschutz Medical Campus, University of Colorado, Boulder, USA, personal communication). Together these data confirm that Refilins are dispensable for oligodendrogenesis but might function in other aspects of the NG2+ cells biology (Nishiyama et al., 2009).

In mesenchymal cells, the Refilin/FLNA complex regulates perinuclear Actin bundles that organize the Actin cap and decrease cell nuclear height (Gay et al., 2011a,b). Three dimensional reconstitution of the Actin cytoskeleton in NG2 cells revealed that these cells do not organize perinuclear Actin caps. Instead NG2 cells have unusually high nuclear heights that are responsible for high cell refringence on phase contrast observations. Importantly, we ensured that ectopic expression of RefilinB-GFP did not promote Actin cap formation in NG2 cells (not shown). Live video imaging of NG2+ cells overexpressing RefilinB-GFP shows an increase in lamellipodia protrusion along and at the tip of dendritic processes, suggesting a new function for Refilin in dynamic cell membrane remodelling (Movies 1-4; Figs 5–7). In neuronal cells, the cellular forces driving protrusion of growth cone depend on specific Actin binding proteins that regulate the transition and/or organization between different states of Actin assembly (He et al., 2015; Nemethova et al., 2008; Pak et al., 2008). At least two specialized Actin bundles control the dynamics of growth cones, the filopodia Actin in the peripheral zone and the Actin arcs localized in the transitional zone (Schaefer et al., 2002). In NG2 cells, RefilinA-GFP accumulates in the transitional zone of the growth cone with no evidence for filopodia labelling at the periphery (Fig. 5C). Moreover, suppression of RefilinB by shRNA decreases the frequency with which lamellipodia form but does not inhibit filopodia (Movie 4). It is likely that in NG2 cells the RefilinB/FLNA complex regulates lamellipodial dynamics by stabilizing Actin bundles and by integrating bundles with the lamellipodia Actin network of the transitional zone. Further support for this model comes from studies with spreading U373MG cells on Fibronectin-coated dishes (Figs 7, 8). When infected with recombinant adenovirus-expressing RefilinA-GFP, U373MG cells showed more rapid spreading on Fibronectin-coated dishes than those infected with control adenovirus (Fig. 7). In spreading U373MG cells, Refilins function as switches to convert Filamins from Actin-branching into Actin-bundling proteins to structure Actin arcs in the lamella (Fig. 8). Direct relationships between the inhibition of Actin-branching activity in cells and the formation of Actin arcs during cell spreading has recently been reported (Henson et al., 2015). Taking into account the very short half-life of Refilins, and the relatively high FLNA concentration in the cells, we hypothesize that the lamellipodial protrusion made visible by overexpression of the Refilins in NG2 and U373MG cells represents one manifestation of the Actin-bundling activity mediated by the Refilin/Filamin complex to regulate the dynamics of cell membrane remodelling. A function for the Refilin/FLNA complex linked to the dynamics of plasma membrane remodelling is consistent with observations made on epithelial NMuMG cells. In polarized epithelial NMuMG cells, TGFβ stimulation promotes transient accumulation of RefilinB protein at the apical cell-cell contact sites, where it co-localizes with FLNA (Gay et al., 2011a). The apical reorganization of the Actin cytoskeleton mediated by the RefilinB/FLNA complex contributes to remodelling of the lateral membrane dynamics and changes in cell-cell interactions that accompany mesenchymal transition (Gay et al., 2011b). An emerging picture from all these studies is that Actin organisation and dynamics depend on the Refilin/Filamin complex functions in both cell-substrate adhesion and cell-cell interactions. In the brain, mutations in the X-linked FLNA gene lead to the periventricular heterotopia that is characterized by a failure in neural progenitors to migrate into the cerebral cortex and the formation of nodules in the sub-ventricular zones (Sheen et al., 2001). Because both defects in cell-cell contacts and migration may contribute to the accumulation of neuroblasts at the ventricular surface in humans with FLNA mutations (Feng et al., 2006), it would be interesting to evaluate whether this is the function of FLNA when complexed to Refilin that is altered in the periventricular heterotopia syndrome. In light of our work on NG2 cells, we suggest that these studies may also prove helpful for future research directions aimed at the specific roles of Refilin proteins in the fate and functions of this intriguing brain cell population (Nishiyama et al., 2015).

## MATERIAL AND METHODS

### Antibodies

Antibodies purchased from commercial suppliers are as follows: FilaminA mouse monoclonal (AbNova, H00002316-M01, used at 1:1000); FilaminA mouse monoclonal (USBiological, F4510, used at 1:1000); NG2 mouse monoclonal (Upstate, 05-710, clone 132.38, used at 1:500); NG2 rabbit polyclonal (Chemicon, ab5320, used at 1:500); GFP mouse monoclonal (Abcam, ab-1218, used at 1:500); Rat anti-Myc tag antibody (JAC6-Serotec, used at 1:500). The mouse monoclonal antibodies agains α-tubulin and β-tubilin were a gift from Dr. Laurence Lafanachère, Institute for Advanced Biosciences, Grenoble, France (used at 1:5000 on western blot and at 1:1000 for IF). Mouse anti-Myc tag, A2B5, O4 hybridomas were amplified in the laboratory. Affinity­-purified chicken and guinea pig antibody against RefilinA and RefilinB were obtained as previously described (Gay et al., 2011b). Anti-Vimentin (Santa Cruz, sc-373717) was used at 1:1000. Alexa Fluor 546 Phalloidin (Invitrogen; A22283) was used at 1:250. Secondary antibodies: conjugated with Alexa Fluor 488 (Molecular Probes), Cyanine3 or Cyanine5 (Jackson ImmunoResearch) were all used at 1:2000. Secondary antibodies coupled to horseradish peroxidase were from Jackson ImmunoResearch (115-035-174, 111-035-144 and 112-035-175) and used at 1:5000.

### Cell cultures

To produce rat OPC cultures, newborn rats of either sex were killed by decapitation, brains were removed and hemispheres were isolated in PBS-6% glucose. Brain cells were dissociated in 30 U/ml papain (Worthington), 0.24 mg/ml L-cysteine (Sigma), 40 µg/ml DNAase (Sigma) treatment for 1 h at 37°C. Cells were centrifuged and resuspended in stop medium [Leibovitz's medium (L15, Gibco), 1 mg/ml ovomucoid (Worthington), 50 µg/ml bovine serum albumin (BSA, Gibco), DNAase]. After centrifugation, cells were mechanically dissociated in L15 and 40 µg/ml DNAase and plated in poly-L-lysine-coated (Sigma) dishes in DMEM-Glutamax-4.5 g/l glucose (Invitrogen)+10% fetal calf serum (FCS). After 10 days, OPC were isolated from microglia and astrocytes by series of plate shaking, and cultured on poly-L-lysine coated dishes (Sigma) in OPC proliferation medium [Neurobasal medium (Gibco), 2% B27 complement (Invitrogen), 2 mM Glutamine (Gibco)] supplemented daily with 0.1 µg/ml bFGF and 10 ng/ml PDGF. Long-term secondary cultures were obtained after 3 months of culture in proliferation medium. For oligodendrocyte differentiation, cells were shifted to medium without bFGF plus thyroid hormone (40 ng/ml) (Tang et al., 2000). Rat neurospheres were obtained as previously described (Balenci et al., 2006). For specific purpose (Fig. 3D), OPC and neural progenitors were cultivated in bacteriological non-coated petri dishes (BD biosciences) as spheres in Neurobasal medium with 2% B27 complement, 2 mM Glutamine and supplemented daily with 0.1 µg/ml bFGF and 10 ng/ml PDGF or 20 ng/ml EGF for two weeks with cell dissociation and passages every 3 days. Cells were collected by centrifugation and lysed in SDS-sample buffer. U373 MG cells were cultured in growth medium: Dulbecco's modified Eagle's medium supplemented with 10% fetal bovine serum (FBS). Cells were free of mycoplasma contamination.

### Isolation of OPC subpopulations by flow cytometry

The following mouse monoclonal primary antibodies were used: NG2 (1:500 dilution), A2B5 (1:10) and O4 (1:3) in an adequate combination to separately harvest NG2^+^/A2B5^−^/O4^−^, NG2^+^/A2B5^+^ and NG2^−^/O4^+^ cells. Primary antibodies were directly added to the OPC proliferation medium of established secondary cultures of OPC, and incubated for 15 min at 37°C. The medium was then removed, and cells were rinse once. New OPC proliferation medium, containing the respective secondary antibodies conjugated with either Alexa Fluor 488 or Cyanine3 was added and incubated 15 min at 37°C. Cells were rinsed once, harvested by gentle pipetting, centrifuged at 100 ***g*** for 5 min and resuspended in PBS. A MoFlo flow cytometer (Dako Cytomation) was used to sort our population of interest and cells were collected in PBS. Sorted cells were finally centrifuged at 100 ***g*** for 5 min and the pellets were immediately frozen in liquid nitrogen before being store at −80°C until RNA extraction.

### RNA extraction and amplification

Total RNA was extracted from frozen cells with the Qiagen RNeasy minikit (Qiagen) following manufacturer's protocol and quantified with Qubit (Invitrogen) using Quant-IT RNA assay (Molecular Probes). Quality of total RNA was verified by microchips on Agilent Bioanalyzer 2100 (Agilent Technologies). Total RNA (10 ng) were amplified and biotin-labelled by two rounds of *in vitro* transcription (IVT) with a Message Amp aRNA kit (Ambion) following the manufacturer's protocol. Before amplification, spikes of synthetic mRNA at different concentrations were added to all samples; these positive controls were used to ascertain the quality of the process. aRNA yield was measured with an UV spectrophotometer and the quality on nanochips with the Agilent 2100 Bioanalyzer.

### Array hybridization and processing

Ten micrograms of biotin-labeled aRNA was fragmented using 5 µl of fragmentation buffer in a final volume of 20 µl, then was mixed with 240 µl of Amersham hybridization solution (GE Healthcare Europe GmbH) and injected onto CodeLink Uniset Rat Whole Genome bioarrays (40K) containing 36,000 rat oligonucleotide gene probes (both from GE Healthcare Europe GmbH). Arrays were hybridized overnight at 37°C at 300 rpm in an incubator. The slides were washed in stringent TNT buffer (0.1 M Tris-HCl, 0.15 M NaCl, 0.05% Tween-20) at 46°C for 1 h, and then a Streptavidin-Cy5 (GE Healthcare) detection step was performed. Each slide was incubated for 30 min in 3.4 ml of Streptavidin-Cy5 solution as described previously (Fevre-Montange et al., 2006), then was washed four times in 240 ml of TNT buffer, rinsed twice in 240 ml of water containing 0.2% Triton X-100, and dried by centrifugation at 50 ***g***.

The slides were scanned using a Genepix 4000B scanner (Axon) and GenePix software (Molecular Devices), with the laser set at 635 mm, the laser power at 100%, and the photomultiplier tube voltage at 60%. The scanned image files were analysed using CodeLink expression software, version 4.2 (GE Healthcare), which produces both a raw and normalized hybridization signal for each spot on the array.

### Microarray data analysis

The relative intensity of the raw hybridization signal on arrays varies in different experiments. CodeLink software was therefore used to normalize the raw hybridization signal on each array to the median of the array (median intensity is 1 after normalization) for better cross-array comparison. The threshold of detection was calculated using the normalized signal intensity of the 100 negative control samples in the array; spots with signal intensities below this threshold are referred to as ‘absent’. Quality of processing was evaluated by generating scatter plots of positive signal distribution. Signal intensities were then converted to log base 2 values. Statistical comparison and filtering were performed using Genespring software 7.0 (Agilent). Selection of genes was performed by pairwise comparisons between NG2+/A2B5−/O4−, NG2+/A2B5+ and NG2−/O4+ cells. Each sample from one group was compared with each sample from the other group and only gene showing a variation change ≥2 were retained. RNA preparation, DNA array hybridization and data analysis have been realized in the genomic and microgenomic platform ProfileXpert. The retained genes of interest were listed and classified according to their functions, on the basis of the Gene Ontology Consortium using Ingenuity Pathway Analysis (Mountain View).

### RNA extraction and reverse transcription

Preparation of total RNA was done using Trizol reagent (Invitrogen). RNA integrity was assessed by agarose gel electrophoresis and the amount of total RNA was determined by UV spectrophotometry. 500 ng of total RNA was used to synthesize complementary DNA using oligo d(T) primers and Superscript II reverse transcriptase (Invitrogen). The complementary DNA was stored at −20°C until use.

### Quantitative PCR (qPCR)

qPCR was performed on the LightCycler2.0 machine (Roche) using LightCycler^®^ FastStart DNA MasterPLUS SYBR Green I mix (Roche) with the following program: 10 min incubation at 95°C, followed by 45 cycles of 95°C for 10 s, 55°C for 10 s and 72°C for 15 s, and a final step of 65°C for 15 s. The *β-actin* or *Gapdh* gene was selected as reference gene for internal standardization. Enrichment (x) was calculated using the following formula: x=2^−ΔΔCt^ (Livak and Schmittgen, 2001). All the data were presented in terms of relative mRNA expressed as mean±s.d.

### Adenovirus infection

RefilinB-targeting shRNA sequences were previously designed from DESIR software and corresponded to shRNA RefilinB lentiviral particle #5 in Gay et al. (2011b). shRNA sequences were synthesized, pair annealed, and subcloned into pQAd vector (ViraQuest). Recombinant adenovirus particles expressing GFP, RefilinA-GFP, RefilinB-GFP and ShRefilinB were obtained from ViraQuest. Cells were incubated with viral particles at multiplicity of infection (MOI) of 15 diluted in growth medium.

### Videomicroscopy

For time lapse microscopy of oligodendrocyte progenitors, cells were grown on poly-lysine coated glass coverslips pre-coated or not with polystyrene, as indicated in legends. Cells were infected with recombinant adenovirus particles expressing GFP, GFP-RefilinB or ShRefilinB as described above. After 20 h post-infection, coverslips were placed inside the videomicroscopy platform equipped with a device enabling regulation of temperature and CO_2_ level. Time lapse images were collected with an inverted microscope (Axiovert, 200M, Zeiss) controlled by Metamorph software (Molecular Devices). Cells were observed with a 60× objective. Phase contrast images were acquired every 5 s for 10 min.

### Immunofluorescent staining

Cells grown on poly-L-lysine coated coverslips were fixed with methanol at −20°C or with 4% paraformaldehyde in phosphate-buffered saline and permeabilized with 0.2% Triton X-100. Cells were incubated with blocking buffer [5% newborn goat serum (NGS)-TBS] and incubated with primary antibodies overnight at 4°C, cells were then washed in TBS and stained with the appropriate secondary antibodies. Images were obtained with a Zeiss (Axiovert 200 M) microscope or with a Leica (TCS SP2) confocal microscope. Hoescht, Alexa Fluor 488, Cyanine3 (Cy3), and Cyanine5 (Cy5) fluorophores were excited and collected sequentially (400 Hz line by line).

### Co-immunoprecipitation

Cells were lysed in 500 µl lysis buffer (40 mM Tris pH 7.5, 150 mM NaCl, 0.3% Triton X-100, 4 mM EDTA, 4 mM EGTA) supplemented with antiproteases and antiphosphatases. Protein G Sepharose beads (Sigma) coupled to mouse anti-Myc antibody were incubated with cell lysate for 2 h at 4°C. Precipitates were washed four times in lysis buffer, solubilised in SDS sample buffer, fractionated by SDS-PAGE and analysed by western blotting.

### Cell treatment for Refilin stability

Cells were treated with cycloheximide (100 µg/ml) for protein translation inhibition or MG132 (5 µM) for proteasome inhibition. Cells were scraped and lysed in lysis buffer supplemented with anti-proteases and anti-phosphatases. 15 µg of total protein extracts were fractionated by SDS-PAGE and analysed by western blotting as described in figure legends.
